# Automatic price appraisals: why they matter and how to measure them

**DOI:** 10.3389/fpsyg.2024.1359007

**Published:** 2024-04-26

**Authors:** Daria Altenburg, Adriaan Spruyt

**Affiliations:** Department of Marketing, Innovation and Organisation, Ghent University, Ghent, Belgium

**Keywords:** automatic cognition, implicit measures, price appraisals, implicit attitude, willingness-to-pay (WTP)

## Abstract

Accurately estimating consumers’ willingness-to-pay (WTP) is crucial to product design, pricing decisions, and the design of competitive marketing strategies. However, traditional self-report measures of WTP are susceptible to many reporting biases, including tactical responding or an inability to make accurate estimates. Importantly, appraisals also occur automatically (i.e., in the absence of substantial time, intention, awareness, and/or substantial cognitive resources) and implicit measures used to capture automatic appraisals are less susceptible to the sort of reporting biases that self-report measures can be affected by. However, the only existing implicit measure for assessing automatic price appraisals (the Task Rule Congruency paradigm, ‘TRC’) is impractical because of the large number of trials and time it requires. Accordingly, here we introduce the Implicit Attribute Classification Task (IMPACT), test its effectiveness for the measurement of automatic price appraisals (Study 1), and directly compare its effectiveness and utility with that of the TRC (Study 2). We find that the IMPACT is an efficient measure of automatic price appraisals, that it produces considerably larger effects compared to the TRC, and that it does so while substantially shortening the procedure. We also discuss how the IMPACT scores can be used to derive an implicit measure of willingness to pay. Our findings make a substantial contribution to both research and practice by providing an effective tool that facilitates, for the first time, an efficient exploration of implicit WTP.

## Introduction

Willingness-to-pay (WTP) refers to the maximum price at which consumers will buy a product or service (Varian, 1992). Accurately gauging this price point is crucial, as WTP measures play a key role in pricing decisions, competitive strategies, and product development. Both direct [e.g., the Van Westendorp Price Sensitivity Meter (Van Westendorp, 1976)] and more subtle (e.g., choice-based conjoint analysis) WTP measures rely on explicit consumer price evaluations or explicitly stated preferences (Breidert et al., 2006; Miller et al., 2011). However, this approach is prone to a number of reporting biases and can lead to inaccurate estimations as consumers may not always want to accurately volunteer this information, may respond strategically, or may perhaps simply be unable to access or express their willingness-to-pay (Podsakoff et al., 2012; Wang and Ge, 2016). For instance, a recent meta-analysis demonstrated that traditional measures of WTP tend to overestimate consumers’ real WTP (Schmidt and Bijmolt, 2020), therefore limiting their utility as ideal tools for price optimisation.

Dezwaef et al. (2019) recently suggested that one way to circumvent these limitations would be to employ a measure that derives willingness-to-pay from automatic processes [i.e., spontaneous evaluative processes that can be characterised as fast, efficient, unintentional, uncontrolled and/or unconscious (Moors and De Houwer, 2006)], as opposed to explicit processes (i.e., deliberated, self-reported evaluations). Specifically, Dezwaef et al. (2019) argued that comparisons between a given price and an internal (typically memory-based) reference point, such as the price of the item at past purchases, occur automatically and that these automatic comparisons inform consumers’ WTP. Accordingly, these automatic comparisons can be leveraged as a starting point to assess automatic price appraisals and in turn arrive at an estimate of implicit WTP.

To provide first proof-of-principle, Dezwaef et al. (2019) used a task-rule congruency paradigm (‘TRC’) to test whether price evaluations occur automatically. The task-rule congruency effect occurs when two tasks share the same stimuli and mode of response (commonly a key-press) and the automatic activation of a response rule that had been learned in one task interferes with performance on a second task (Kiesel et al., 2010; Liefooghe et al., 2012). Specifically, performance is facilitated if the active response rule and the irrelevant response rule map onto each other (i.e., are compatible) and hindered if the active response rule and the irrelevant response rule do not map onto each other (i.e., are incompatible) (Meiran and Kessler, 2008). For instance, participants may be presented with a set of word-stimuli that differ in terms of colour (green vs. yellow) and font style (italic vs. bold). A first task may require the stimuli to be classified according to colour, whereas a second task may require the stimuli to be classified according to font style. In both tasks, participants classify the stimuli by pressing either a left-hand key (for ‘green’ in task one and ‘italic’ in task two), or a right-hand key (for ‘yellow’ in task one and ‘bold’ in task two). Because each response key now shares two meanings (e.g., ‘green’ and ‘italic’ for the left-hand key), response speed will be facilitated if the response rule of the irrelevant task is compatible with the response rule of the relevant task. Based on this logic, Dezwaef et al. (2019) tested whether an evaluative response to prices is automatically activated during a second, non-evaluative categorisation task, even if the evaluative response rule is no longer relevant to the task instructions.

To demonstrate this principle, Dezwaef et al. (2019) tested whether they could use this method to record known pricing evaluations of everyday grocery items. Given consumers’ familiarity with grocery prices [product familiarity was also confirmed in a pre-test by Dezwaef et al. (2019)], manipulating the prices of these items to be either cheaper or more expensive than retail prices should elicit a corresponding response in the participants. Specifically, the prices were manipulated across seven intervals (−70, −40%, −10%, 0%, +10%, +40%, +70%). In line with this manipulation, if the measure is working as expected, the recorded attitudes should follow a linear trend aligned with these intervals.

In the task-rule congruency paradigm used by Dezwaef et al. (2019), the procedure consisted of two consecutive single-task phases. First, in an evaluative phase, participants were required to evaluate a series of product-price combinations as ‘cheap’ or ‘expensive’ by pressing a left-hand key (cheap) or a right-hand key (expensive). This phase was used to train participants to map the evaluations ‘cheap’ or ‘expensive’ onto the corresponding keys. Next, participants encountered a categorisation phase, that again presented a series of product-price combinations. Using the same keys as in the evaluation phase, participants were instructed to categorise the price stimuli based on parity (Study 1) or font style (Study 2), and no longer evaluate the prices as cheap or expensive. During this phase, it was expected that the (now irrelevant) task rule learned in the evaluation phase would interfere with the performance on the categorisation task. Specifically, if the required categorisation response was compatible with the evaluative response, response speed and accuracy were expected to be facilitated. In contrast, if the required categorisation response were incompatible with the evaluative response, response speed and accuracy were expected to be hindered.

Dezwaef et al. (2019) predicted that large discrepancies between the tested price and the internal reference point (here: common retail prices) would lead to greater task-rule congruency interference compared to small discrepancies. In line with this prediction, they observed that the task-rule congruency effects were larger when the tested price was unambiguously more expensive or unambiguously cheaper compared to the participants’ internal reference point, and that the effects were smaller the closer the tested price approached the internal reference point. These findings by Dezwaef et al. (2019) are a potentially valuable first demonstration of the automatic processing of price evaluations. However, Dezwaef et al. (2019) emphasized the need for a shorter and less resource-intensive procedure in order for this approach of measuring price appraisals to be easily used in further research and applied to marketing practices.

Following this advice, we developed a new measure of automatic price appraisals based on the Implicit Attribute Classification Task (“IMPACT”, Altenburg and Spruyt, 2022). Similar to the TRC paradigm The IMPACT is an implicit measure that draws on task-rule congruency principles to assess automatic relational processing of stimulus pairs. Specifically, each trial of the standard IMPACT consists of the presentation of a stimulus compound comprising a target (e.g., fries) and an attribute (e.g., healthy) followed by one of three task cues that indicates either an evaluative (cue:???) or non-evaluative (cues: YES, NO) task-rule. During the evaluative trials, participants are required to indicate whether the attribute applies to the target by pressing either a left-hand or right-hand key. During the non-evaluative trials, participants are required to press the left-hand or right-hand key depending on whether the cue “YES” or “NO” appears, that is, regardless of their evaluation of the match between attribute and target. The evaluative and non-evaluative task trials are presented in a mixed-task block. Because the task rule changes but the response keys stay consistent, there are two meanings mapped onto each of the response keys (e.g., the cue “YES” and ‘the attribute does apply to the target’ for the left-hand key; and the cue ‘NO’ and ‘the attribute does not apply to the target’ for the right-hand key). Due to this shared mapping, response speed and accuracy will be facilitated if the required response in the non-evaluative trials (e.g., press left for the cue “YES”) is congruent with the response rule learned in the evaluative trials (e.g., press left if the target is healthy). In turn, if the required response in the non-evaluative trials is incongruent with the response rule learned in the evaluative trials, response speed and accuracy will be obstructed. Following this procedure, automatic appraisals of a target can be derived from response latencies and accuracy rates. Importantly, the flexible trial composition allows the IMPACT to be adapted for a wide range of applications, such as in this specific context assessing the relationship between a product and a range of prices.

There are several reasons to predict that an adapted version of the IMPACT would outperform the procedure used by Dezwaef et al. (2019). First, the evaluative trials and non-evaluative trials are presented in an intermixed, random order instead of a sequential, blocked order [as used by Dezwaef et al. (2019)]. Previous research has demonstrated that the task-rule congruency effect on reaction times is commonly larger in task-switching designs such as that used in the IMPACT, compared to single-task blocks [as used by Dezwaef et al. (2019)] (Meiran, 2000; Meiran, 2005). Second, previous research using the IMPACT has demonstrated its effectiveness in recording automatic appraisals based on only a limited number of trials (Altenburg and Spruyt, 2022), suggesting that the IMPACT will likely be a less resource-intensive procedure than that by Dezwaef et al. (2019). Lastly, the IMPACT is simple to use in an online setting and therefore practical to target a wide range of specific consumer segments. Dezwaef et al. (2019) emphasized that, ideally, an implicit measure of willingness to pay should be suitable for testing online because it would allow researchers to more easily reach a wider range of specific consumer segments, and pointed out that the procedure used in their study may be too time-consuming to be an effective online tool. Accordingly, we propose an adapted version of the IMPACT, and, closely following the methods put forward by Dezwaef et al. (2019), assessed its effectiveness as an implicit measure of automatic price appraisals.

### Outline of the pricing-IMPACT

**Figure 1:**
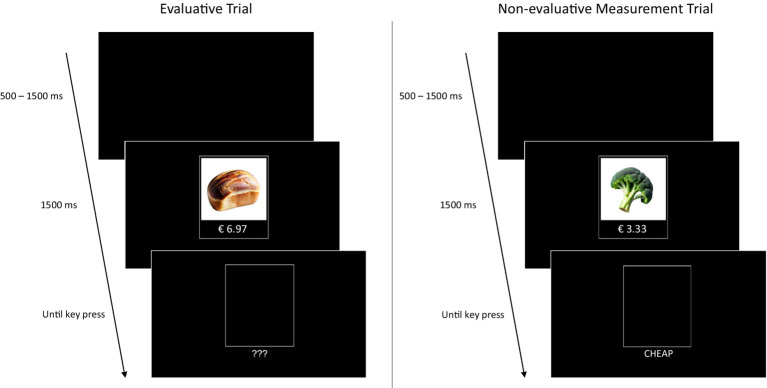
Trial types presented in the pricing IMPACT. Trials are presented in randomised, intermixed order. Both trial types start with a black screen with a random presentation time between 500 and 1,500 ms, followed by a product-price pairing that disappears after 1,500 ms. Following the product-price pairing, a task cue is presented until a key press is registered. The Evaluative trials (left) are indicated by three question marks and participants are asked to press a key to indicate whether the product-price pair is cheap or expensive. These trials are used to train participants to map the evaluations “cheap” and “expensive” onto the respective keys. In non-evaluative categorisation trials (right), participants are asked to press the left-hand or right-hand key depending on whether the cue ‘CHEAP’ or the cue ‘EXPENSIVE’ appears, regardless of their evaluation of the product-price pair. These trials are used to infer implicit attitudes based on interference effects. Note that, due to potential copyright concerns, Figure 1 illustrates the IMPACT procedure using images of generic (i.e., unbranded) products. The actual stimuli used in Experiment 1 are publicly available at https://osf.io/w4kvz/.

In each trial of the pricing IMPACT, participants are briefly presented with a product-price compound that disappears after 1,500 ms, followed directly by a task cue. Because the IMPACT consists of intermixed evaluative trials and non-evaluative categorisation trials, the task cue is used to indicate to participants which task they are required to perform. In evaluative trials (indicated by the cue “???”), participants are asked to indicate whether the presented product-price compounds are cheap or expensive by pressing either a left-hand or a right-hand key. These trials are used to train participants to map the evaluations “cheap” and “expensive” onto the respective keys and are not used for analysis. In non-evaluative categorisation trials, participants are asked to simply press the left-hand or right-hand key depending on whether the cue ‘CHEAP’ or the cue ‘EXPENSIVE’ appears, regardless of their personal evaluation of the product-price combination (see Figure 1 for a schematic of the procedure). These trials are used to infer implicit attitudes based on task-rule congruency effects (i.e., the interference of the response rule learned in the evaluative trials with performance on the non-evaluative categorisation trials, see p. 2–3).

Although the non-evaluative categorisation task does not require participants to process the product-price compounds (as the response is dependent only on the cue), the relational processing of the product and price compounds is promoted across all trials because participants cannot anticipate whether an evaluative or non-evaluative task cue will appear. Accordingly, response times are expected to be fast if the evaluative and non-evaluative response are compatible and slow if the evaluative and non-evaluative response are incompatible.

## Experiment 1

Experiment 1 tested the effectiveness of the pricing IMPACT at recording automatic price appraisals. Modeling this experiment closely on the materials put forward by Dezwaef et al. (2019), we tested automatic price appraisals of common supermarket products on prices ranging from unambiguously cheap to unambiguously expensive.

### Method

#### Ethical statement

This study complied with the Research Code of Ethics of the Faculty of Economics and Business Administration, Ghent University. Participants were informed that their participation was voluntary, that they could end their participation at any time, and that their data would be processed, stored, and reported anonymously. Participants then gave written informed consent prior to participation.

#### Participants

One hundred forty-two volunteers initially took part in the study. All data was collected between May 1st and May 18th, 2022. Recruitment took place online and relied on a Belgian convenience sample. Eight participants started or completed the task more than once. In these cases, only the first participation data was retained and subsequent repetitions discarded. Six respondents did not proceed past the instructions, and another thirty-five respondents did not complete the full experiment and were excluded from analysis. Four participants were excluded because of excessive error rates in the IMPACT (43.75–53.57%). Additionally, eight participants were excluded as an excessive percentage of their trials had to be replaced due to error rates and extreme response latencies taken together (41.96–50.89%). We defined error rates and trial exclusions of 41.96% and above as excessive. This was, in line with previously reported data exclusion standards of the authors, based on the binomial probability of observing a given number of correct responses if a respondent was actually performing at chance level. One further participant was excluded because their mean response speed (1,465 ms) deviated more than 2.5 SD from the samples’ mean response speed (cut-off value: 1396 ms). A final sample of *N* = 88 was retained for analysis (45 women, 41 men, 2 did not report their gender; *M_age_* = 35.72 years, *Mdn*_age_ = 28.5 years, *Range*_age_ = 20–75 years).

### Materials and procedure

#### Stimuli and price points

The product stimuli used in this study were the same thirty-six products selected by Dezwaef et al. (2019) based on a pre-test using a Belgian sample to ensure that participants would be familiar with the products and average retailing price. Also in line with Dezwaef et al. (2019), twenty-eight of these thirty-six products were shown during the evaluation trials and eight were shown in the non-evaluative measurement trials. To ensure that participants would learn the required evaluative mapping of cheap and expensive during the evaluative trials, the price points shown with the products in these trials were calculated as unambiguously expensive (70% above the current retail price) or unambiguously cheap (70% below the current retail price, retail prices as indicated on the website of Carrefour Belgium, data retrieved on April 7^th^, 2022). In the non-evaluative measurement trials, the prices varied across seven price points, ranging from unambiguously cheap to unambiguously expensive. Specifically, for each product we calculated six price points (−70, −40%, −10%, +10%, +40%, +70%) based on and in addition to the actual retail price (0%). The exact stimuli and price points used in this study are publicly available at https://osf.io/w4kvz/.

#### Procedure

Participants were able to access the study via desktop devices. Participation on mobile devices was not possible. Participants were first asked about their age, gender, occupation, average net income, and frequency of grocery shopping. Next, they were forwarded to the pricing IMPACT.

#### Pricing IMPACT

The pricing IMPACT was built using PsychoPy software (Peirce et al., 2019) and administered via the PsychoPy launching platform Pavlovia.org. Participants were instructed to use the E-key and the I-key to 1) evaluate the shown product-price combination as cheap or expensive if the???-cue appeared, or 2) categorise the cue words CHEAP and EXPENSIVE, regardless of their personal evaluation. The response assignment of the E- and the I- key was counterbalanced. For half of the participants the evaluation ‘cheap’ and the cue word CHEAP were mapped onto the E-key, whereas the evaluation ‘expensive’ and the cue word EXPENSIVE were mapped onto the I-key. The reverse mapping was true for the other half of participants. Participants were encouraged to respond as quickly as possible while keeping a low error rate. To familiarise participants with the task, they first completed eight practice trials, followed by a reminder of the instructions.

Next, participants completed a measurement block that consisted of the intermixed evaluative trials and non-evaluative measurement trials. For the evaluative trials, each of the twenty-eight products was displayed once with the unambiguously high price, and once with the unambiguously low price, resulting in a total of 56 evaluative trials. For the non-evaluative measurement trials, each of the eight product and price-point combination was displayed once with the cue “CHEAP” and once with the cue “EXPENSIVE” resulting in a total of 112 non-evaluative measurement trials. Altogether, participants completed one measurement block, i.e., a total of 168 trials presented in randomised order. The inter-trial interval was set to randomly vary between 500 ms and 1,500 ms. The product-price compounds were always presented for 1,500 ms before they disappeared and one of the task cues appeared. The task cue was always displayed until a key press was registered. In both the evaluative and non-evaluative trials participants were presented with a red “X” if a wrong key press was registered. This error feedback remained on screen until the respondent corrected their response.

### Results

The data were prepared and aggregated in the RStudio environment version 2022.07.1.554 (R Studio Team, n.d.) using R version 4.1.2 (2021-11-01) (R Core Team, 2021) and the packages tidyverse 1.3.1 (Wickham et al., 2019), gtools 3.9.2. (Warnes et al., n.d.), and stringr 1.4.0 (Wickham, n.d.). Analyses were carried out using SPSS IBM SPSS Statistic for Windows, Version 27. The data of this experiment are publicly available at https://osf.io/w4kvz/.

#### Data preparation

For the calculation of the D scores, only the critical trials (i.e., categorisation trials with the cues “EXPENSIVE” or “CHEAP”) were used. All data preparation decisions were based on consistent standards applied by the authors (Altenburg, 2023) and empirically derived recommendations described by Greenwald, Nosek, and Banaji (Greenwald et al., 2003). Trials with extreme response latencies (below 300 ms or above 5,000 ms) were winsorised, so that trials with a response latency below 300 ms were replaced with 300 ms, and trials with a response latency above 5,000 ms were replaced with 5,000 ms. In line with recommendations by Greenwald et al. (2003), error trials were excluded and instead replaced by the participant’s mean response latency based on correct trials plus a penalty of 600 ms. One D score for each price point was then calculated following the standard D score formula by Greenwald et al. (2003). This formula divides the differences in mean response latencies on compatible and incompatible trials by their pooled standard deviation. Because compatibility is reversed for negative and positive prices in this study (e.g., the cue “CHEAP” is compatible with price points below the retail price, but incompatible with price points above the retail price), we reversed compatibility scoring at the 0% price point. That is, to preserve a uniform directionality of the D scores, for each price point we subtracted the mean response latency on trials with the cue “EXPENSIVE” (i.e., trials that were incompatible for all price points below the retail price, and compatible for all price points above the retail price) from the mean response latency on trials with the cue “CHEAP” (i.e., trials that were compatible for all price points below the retail price, and incompatible for all price points above the retail price) and divided this by the pooled standard deviation. This always resulted in a D score below zero if a price was appraised as cheap, and in a D score above zero if a price was appraised as expensive. Following this procedure, we derived one D score for each of the seven price points.

#### Internal consistency

We estimated an overall internal consistency score using Spearman-Brown corrected mean split-half correlations (Pronk et al., 2022). For each participant, the compatible trials and the incompatible trials were each split into random halves. We calculated one D score using the first halves of the compatible and incompatible trials, and a second D score using the second halves of the compatible and incompatible trials. Next, the D scores of the two halves were correlated at the group level. This process was repeated one hundred times, and a consistency score was derived from the mean correlation using the Spearman-Brown prediction formula. This resulted in an internal consistency estimate of *R_sb_* = 0.42.^1^

#### Evaluative trials

On average, participants identified 88% of the unambiguously cheap and 83% of the unambiguously expensive prices correctly during the evaluative trials. This suggests that respondents understood the evaluative task instructions and were largely able to identify unambiguous prices.

#### Categorisation trials

On average, participants responded correctly on 92% of all categorisation trials (95% of compatible trials, 89% of incompatible trials) during the categorisation trials. This suggests that respondent understood the non-evaluative categorisation task instructions.

#### Pricing appraisals

D scores below zero indicated that a price was appraised as cheap, whereas D scores above zero indicated that a price was appraised as expensive. Moreover, the greater the distance between the tested price and the reference point, the greater the absolute value of the score. Accordingly, if the IMPACT recorded the expected automatic price appraisals, the observed pattern of the D scores should describe a positive linear trend from the lowest negative value at the −70% price point to the highest positive value at the +70% price point, whereby the reference point (0%) should be close to zero.

To test for this pattern of results, we analysed the price-point D scores using a one-way repeated measures ANOVA with seven levels. Mauchly’s test indicated that the assumption of sphericity was violated (*p* < 0.001), therefore a Greenhouse–Geisser correction was applied. Results showed that there was a statistically significant effect of price point, *F*(4.9, 429.1) = 34.30, *p* < 0.001, *η_p_^2^* = 0.28. As shown in Table 1, the mean D scores followed the expected positive linear trend, whereby the lowest negative score was observed at the −70% price point, an almost neutral score was observed at the 0% price point, and the highest positive value was observed at the 70% price point. Trend analysis confirmed that this was a statistically significant linear trend, *F* (1, 78) = 106.02, *p* < 0.001, *η_p_^2^* = 0.55.

**Table 1:** Descriptive statistics for the automatic price appraisal (D scores) by pricing points.

	Price point						
	−70%	−40%	−10%	0%	+10%	+40%	+70%
Mean	−0.50	−0.26	0.05	0.04	0.26	0.37	0.47
SD	0.63	0.53	0.62	0.59	0.54	0.60	0.50

### Discussion

The findings of Experiment 1 suggest that price appraisals occur automatically and that the IMPACT is a suitable tool to capture these automatic price appraisals. Interestingly, we found that participants perceived both the current retail price (0%) and the ambiguously cheap price (−10%) as virtually identical, and (almost) neutral. This is particularly interesting because (1) increasing the price by 10% did invoke a considerable difference in the automatic price appraisal, suggesting that a 10% increment is generally large enough to provoke a measurable change, and (2) the period shortly prior to the data collection, starting in late 2021, was characterised by a worldwide steep increase in inflation rates, with the Belgian Consumer Price Index Inflation having increased by 8.97% at the time of data collection (May 2022), compared to the previous year (Triami Media BV, 2023). The finding that the −10% price point was perceived as neutral, rather than cheap, is consistent with this rapid price increase, potentially suggesting that relatively recent prices, although now out of date, may still hold some influence as an internal reference point.

Importantly, the observation that the IMPACT was able to (1) record the automatic price appraisals, (2) produce high effect sizes, and (3) considerably shorten the task procedure compared to that of the TRC (the exact TRC procedure will be described in Experiment 2) demonstrates the added value and advantages of the IMPACT over the TRC in its own right. To directly investigate this issue, in Experiment 2 we tested how the pricing IMPACT performs in a direct comparison with the TRC paradigm used by Dezwaef et al. (2019).

## Experiment 2

**Figure 2:**
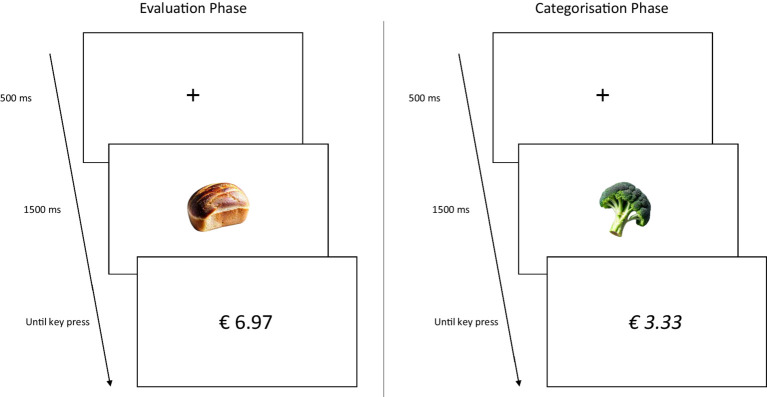
Example of trials in the evaluative and categorisation phase presented in Dezwaef et al. (2019) TRC paradigm. Participants first completed the evaluation phase. Trials started with the presentation of a fixation cross (500 ms), followed by a product. After 1,500 ms, the product disappeared and a price was presented until a key press was registered. In the evaluative phase, participants were asked to indicate as fast as possible whether they evaluated the product as cheap (e.g., press the E-key) or expensive (e.g., press the I-key). Next, participants completed the categorisation phase. The trial composition was identical to that in the evaluation phase, however, participants were now asked to indicate as fast as possible whether the price was printed in italic (e.g., press the E-key) or regular (e.g., press the I-key) font. For both phases, the keys used to respond remained the same. Note that, due to potential copyright concerns, Figure 2 illustrates the TRC procedure using images of generic (i.e., unbranded) products. The actual stimuli used in Experiment 2 are publicly available at https://osf.io/w4kvz/.

In order to directly compare the IMPACT with the TRC, we replicated the method and procedures from Experiment 1 using a between subjects design in which participants completed either the IMPACT or the TRC. Dezwaef et al. (2019) used two different categorisation tasks whereby participants were required to categorise prices based on parity in Study 1, and based on font (italic/regular) in Study 2. Because the second study produced a larger effect, we used the version of the TRC in which the categorisation task was based on the font of the price. Additionally, we shortened the relatively long fixation (from 1,000 ms to 500 ms) and stimulus onset asynchrony (from 2,500 ms to 1,500 ms) used by Dezwaef et al. (2019) to more closely reflect the parameters used in the IMPACT (The IMPACT has no fixation but an inter trial interval that randomly varies between 500 ms – 1,500 ms, and a stimulus onset asynchrony of 1,500 ms). These choices were made deliberately in order to (1) maximise power for the TRC and (2) avoid confounding the potential effect of measurement type with that of differing fixation and stimulus timing. Figure 2 depicts a schematic of the TRC procedure.

### Method

#### Ethical statement

This study complied with the Research Code of Ethics of the Faculty of Economics and Business Administration, Ghent University. Participants were informed that their participation was voluntary, that they could end their participation at any time, and that their data would be processed, stored, and reported anonymously. Participants then gave informed consent prior to participation.

#### Participants

Two hundred nineteen students of the Department of Marketing, Innovation, and Organisation at Ghent University took part in the study. Recruitment took place online. All data was collected between March 28st and March 31st, 2023. Participation was compensated with course credits and the maximum sample size was determined by the number of students enrolled in the modules relevant to the course credit. Eleven participants completed the task more than once. In these cases, only the first participation data was retained and subsequent repetitions discarded. Additionally, in the TRC condition, two participants were excluded as their mean response latencies (1,592 ms and 2,553 ms) deviated more than 2.5 SD from the condition’s mean latency (cut-off value: 1335 ms). One participant was excluded as an excessive percentage of their trials had to be replaced due to error rates and extreme response latencies taken together (52.68%). We defined trial exclusions of 44.64% and above as excessive, based on the probability of observing a given number of correct responses if a respondent was actually performing at chance level. In the IMPACT condition, one participant was removed as their mean response latency (1825 ms) deviated more than 2.5 SD from the condition’s mean latency (cut-off value: 1029 ms). A sample of *N* = 215 (*n_TRC_* = 104, *n_IMPACT_* = 111) was retained for analysis (102 women, 113 men; *M_age_* = 20.5 years, *Mdn*_age_ = 20 years, *Range*_age_ = 19–25 years).

### Materials and procedure

The stimuli and procedure used in Experiment 2 were identical to that in Experiment 1 with the exception that (1) participants were assigned at random to complete either the IMPACT or TRC, (2) the prices were adjusted to reflect inflation in retail prices (retail prices as indicated on the website of Carrefour Belgium, data retrieved on March 1^st^, 2023), and (3) six product stimuli (four in the evaluation phase, two in the categorisation phase) were replaced with items similar to those used in Experiment 1 because at the time of testing, the original items were discontinued by the retailer. The exact stimuli and price points used in this study are publicly available at https://osf.io/w4kvz/.

#### IMPACT

The IMPACT procedure was identical to that described in Experiment 1.

#### TRC

Participants first completed a block of the evaluative trials. Following the procedure used by Dezwaef et al. (2019), each of the twenty-eight products used in the evaluation phase was displayed twice with the unambiguously high price (+70%), and twice with the unambiguously low price (−70%), resulting in a total of 112 evaluative trials (presented in a random order). Next, the categorisation phase consisted of two separate blocks. In each block, each combination of the eight products used in the categorisation phase and the seven price-points was shown in randomised order once with the price printed in italics, and once printed upright. Additionally, a total of eight “refresher trials” (i.e., trials in which the participants were instructed to respond according to the task rule learned in the evaluation phase) were presented pseudo-randomly in each quarter of the two blocks. This procedure resulted in a total of 232 trials in the categorisation phase, divided across two separate blocks. Taken together, participants completed a total of 344 trials across the evaluation and categorisation phase.

### Results

The data were prepared and aggregated in the RStudio environment version 2022.07.1.554 (R Studio Team, n.d.) using R version 4.1.2 (2021-11-01) (R Core Team, 2021) and the packages tidyverse 1.3.1 (Wickham et al., 2019), gtools 3.9.2. (Warnes et al., n.d.), and stringr 1.4.0 (Wickham, n.d.). Analyses were carried out using SPSS IBM SPSS Statistic for Windows, Version 27. The data of this experiment are publicly available at https://osf.io/w4kvz/.

#### Data preparation

For the calculation of the D scores only the critical trials were used (i.e., the categorisation trials for the IMPACT, and the categorisation phase trials for the TRC). Data preparation for both the IMPACT and the TRC was identical to that described in Experiment 1.

#### Internal consistency

The procedure used to estimate internal consistency was identical to that used in Experiment 1. For the IMPACT, this resulted in an overall internal consistency estimate of *R_sb_* = 0.36. For the TRC, this resulted in an overall internal consistency estimate of *R_sb_* = 0.20.^2^

#### Evaluative trials

In the IMPACT, participants identified 88% of the unambiguously cheap and 88% of the unambiguously expensive prices correctly during the evaluative trials. In the TRC, participants identified 94% of the unambiguously cheap and 93% of the unambiguously expensive prices correctly during the evaluative trials. This suggests that in both tasks, respondents understood the evaluative task instructions and were largely able to identify unambiguous prices.

#### Categorisation trials

In the IMPACT, participants responded correctly on 92% of all categorisation trials (96% of compatible trials, 89% of incompatible trials) during the categorisation trials. In the TRC, participants responded correctly on 93% of all categorisation trials (94% of compatible trials, 92% of incompatible trials) during the categorisation trials. This suggests that respondent understood the non-evaluative categorisation task instructions.

#### Pricing appraisals

For both the IMPACT and the TRC condition, we analysed the data using a repeated measures ANOVA, each with D scores as the dependent variable and price point as the within-subject factor. Because Mauchly’s test indicated that the assumption of sphericity was violated in the TRC condition, a Greenhouse–Geisser correction was applied.

Mean D scores for both the IMPACT and TRC increased from the lowest price point to highest price point (see Table 2), whereby this pattern was more pronounced in the IMPACT condition compared to the TRC condition. The results of the ANOVA confirmed a statistically significant effect of price point for the IMPACT, *F* (6, 660) = 94.80, *p* < 0.001, *η_p_^2^* = 0.46. Trend analysis confirmed that there was a statistically significant linear trend, *F* (1, 110) = 357.46, *p* < 0.001, *η_p_^2^* = 0.77. For the TRC, the ANOVA did not detect a significant effect of price point, *F* (6, 554.25) = 1.42, *p* = 0.20, *η_p_^2^* = 0.01.

**Table 2:** Descriptive statistics for the automatic price appraisal (D scores) by pricing points.

	Price point						
	−70%	−40%	−10%	0%	+10%	+40%	+70%
				IMPACT			
Mean	−0.74	−0.39	0.03	0.13	0.23	0.48	0.56
SD	0.50	0.55	0.54	0.49	0.56	0.52	0.52
				TRC			
Mean	−0.11	−0.07	0.05	0.00	−0.02	0.01	0.01
SD	0.54	0.41	0.38	0.40	0.46	0.40	0.41

**Figure 3:**
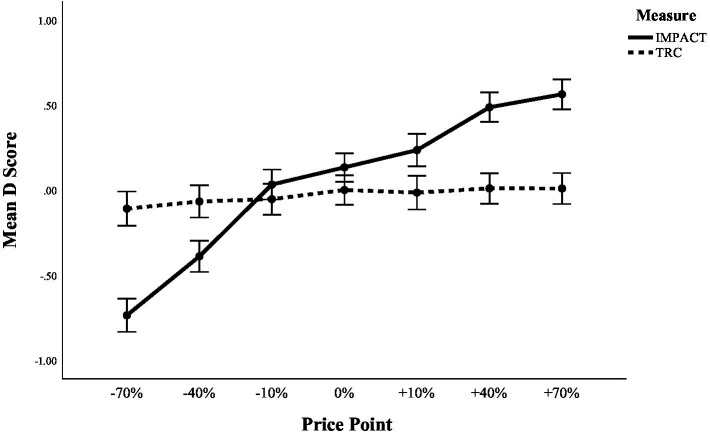
Mean D scores across price points in the IMPACT and the TRC. Error bars = 95% CI.

To directly test whether the effect of price point differed significantly between tasks, we ran an additional analysis in which data from both tasks were entered into one 2 (task) x 7 (price point) ANOVA, with task (IMPACT versus TRC) included as an additional between subject factor. The task by price point interaction was significant in this analysis, *F* (5.56, 1197.29) = 46.96, *p* < 0.001, *η_p_^2^* = 0.18. This pattern of results is illustrated in Figure 3.

### Discussion

The findings of Experiment 2 directly replicate the pattern of results found in Experiment 1, and demonstrate that the IMPACT is an effective tool for capturing automatic price appraisals. Similar to Experiment 1, in Experiment 2, we observed an inflection point in the linear trend at the −10% price point. However, there are some differences in the findings of Experiment 1 and 2 that are worth noting here.

In Experiment 1, both the −10 and 0% price points exhibited relatively neutral, virtually identical D scores (0.05 and 0.04, respectively), indicating that participants did, on average, not discern between the two price points and that both were appraised as relatively neutral. In line with this observation, we noted that this inflection aligned with a rapid (almost) 10% price increase in Belgian Consumer Price Index Inflation (Triami Media BV, 2023), potentially suggesting that recent prices, in addition to the current prices and despite being outdated, could still hold some influence as an internal reference point.

In Experiment 2, we encounter a similar inflection at the −10% price point, yet the −10 and 0% price points demonstrate a notable difference in D scores. Here, we observe a consistent increase in D scores from −10% (0.03) to 0% (0.13) to +10% (0.23). Although this follows a linear trend, this trend line is considerably flatter and the changes in D scores are considerably smaller compared to those observed between the larger increments such as +/− 40% and +/− 70%. This disparity is logical given that the −/+10% increments are considerably smaller. Accordingly, interrogating how sensitive the IMPACT is to small pricing intervals would be an important direction for future research. We again revisit this point in the general discussion.

Importantly, the IMPACT produced effects that were considerably larger compared to the TRC and responses on the IMPACT showed higher internal consistency compared to the TRC. Indeed, although the TRC here was designed specifically in a way to maximise its power to detect participants’ automatic price appraisals, it did not detect significant differences across the different price points.

## General discussion

Across two experiments, we assessed the effectiveness of the IMPACT as an implicit measure of automatic price appraisals. To investigate this issue, we followed the procedure outlined by Dezwaef et al. (2019) and tested automatic price appraisals of familiar products when the price was *unambiguously* more expensive (+40% and + 70%) or cheaper (−40% and − 70%), and when the price was *ambiguously* more expensive (+10%) or cheaper (−10%) compared to participants’ reference point (i.e., current retail price). Using the IMPACT, the results of both Experiment 1 and 2 demonstrated (1) the reference point was appraised as virtually neutral, (2) unambiguously cheap prices were automatically appraised as cheap and unambiguously expensive prices were automatically appraised as expensive, and (3) ambiguously cheap prices and expensive prices were centred closer around the reference point compared to the unambiguous price points. While these findings align very closely with those reported by Dezwaef et al. (2019) and suggest that the IMPACT is a suitable procedure for measuring automatic price appraisals, we were unable to replicate the same pattern of results using the TRC. It should be noted here that the TRC used in Experiment 2 differed from that used by Dezwaef et al. (2019) in two ways. Specifically, we shortened both the fixation timing and the stimulus onset asynchrony to match those used by the IMPACT in order to make the two paradigms comparable (i.e., to avoid confounding measurement type with fixation timing and stimulus onset asynchrony). Importantly, however, these changes to the TRC are unlikely to explain why the TRC did not show a significant effect in our experiment. The IMPACT and TRC are both underpinned by the same task-rule congruency principles and the findings we obtained with the IMPACT demonstrate that the timings we used were sufficient for participants to process the stimuli effectively.

To ensure that participants learned the evaluative task rule, participants in Dezwaef et al. (2019) study completed a total of 112 trials in the evaluative phase. In the categorisation phase of Dezwaef et al.’s study, each product-price combination was presented four times (a total of 224 trials). Thus, the procedure used by Dezwaef et al. (2019) included a total of 336 trials and was reported to last approximately 30 minutes. Using the IMPACT, we were able to considerably shorten this procedure and demonstrate the expected pattern of results with a total of 176 trials (8 practice trials, 168 test trials). On average, this procedure took participants just under 10 minutes to complete. Dezwaef et al. (2019) noted that assessing automatic price appraisals using their methods may be impractical because it is time-intensive and requires a large number of trials. Our data suggest that using the IMPACT would allow both the time and the number of trials required to assess automatic price appraisals to be reduced considerably. Importantly, it has been demonstrated that many effects in reaction time experiments diminish with practice (Miller, 2023). It is therefore possible that the strength of the task rule congruency effect in the TRC might have been declining with increasing practice, and that the TRC might perform better (i.e., record more pronounced differences in D scores between the individual price points) if less trials were considered. To test this, we calculated D scores for the TRC based only on the first half of critical trials (resulting in the inclusion of 112 trials, i.e., the same number of critical trials that were also used in the IMPACT) and repeated the analyses. We found that the differences in D scores we observed using half the trials of the TRC became even less pronounced compared to our results reported in Experiment 2 (Experiment 2: *p* = 0.20, *η_p_^2^* = 0.01 | 50% of trials: *p* = 0.57, *η_p_^2^* = 0.008).

Dezwaef et al. (2019) emphasized that, ideally, an implicit measure of willingness to pay should be suitable for testing online (i.e., can be completed remotely via the internet) because it would allow researchers to more easily reach a wider range of specific consumer segments. Our study took place online and produced results that closely align with those of Dezwaef et al. (2019) laboratory study. Thus, our findings suggest that the IMPACT is not only an effective tool for collecting automatic pricing appraisals that requires fewer trials than the method used by Dezwaef et al. (2019), but it is also suitable for online collection of automatic pricing appraisals. Although we report that forty-one participants did not complete Experiment 1, it is unlikely that this high drop-out rate was a consequence of the IMPACT procedure being too lengthy, as twenty-one of the participants who dropped out of the study did so before completing the eight practice trials. Instead, we suggest that the absence of an incentive for participation may have affected participants’ motivation to complete the study. In line with this reasoning, in the presence of an incentive all participants completed Experiment 2. Thus, we suggest that the IMPACT may be a useful tool for collecting automatic pricing appraisals online, addressing the need for such a measure previously highlighted by Dezwaef et al. (2019).

Finally, it should be noted that, although responses the IMPACT showed higher internal consistency than those on the TRC, the internal consistency estimates we report for the IMPACT are lower than those typically seen for self-report measures. This issue is not unique to the IMPACT (i.e., it is also an issue seen with other implicit measures) and the usefulness of applying the same benchmarks for internal consistency that are used in work using explicit measures to responses on implicit measures has previously been called into question (Bosson et al., 2000; Teige et al., 2004; Gawronski and De Houwer, 2014; Meissner et al., 2019). However, we acknowledge that improving the internal consistency of responses on the IMPACT can only have benefits for researchers who use it. One way to improve the internal consistency of the IMPACT would be to increase the number of observations for each product and price combination. This could be achieved without making the test longer by, for example, reducing the number of individual price points assessed within one test. Regardless of the issue of internal consistency, that the IMPACT scores related to the price points in a meaningful way and produced large effect sizes in both experiments clearly demonstrates the validity of the pricing IMPACT for measuring automatic price appraisals.

**Figure 4:**
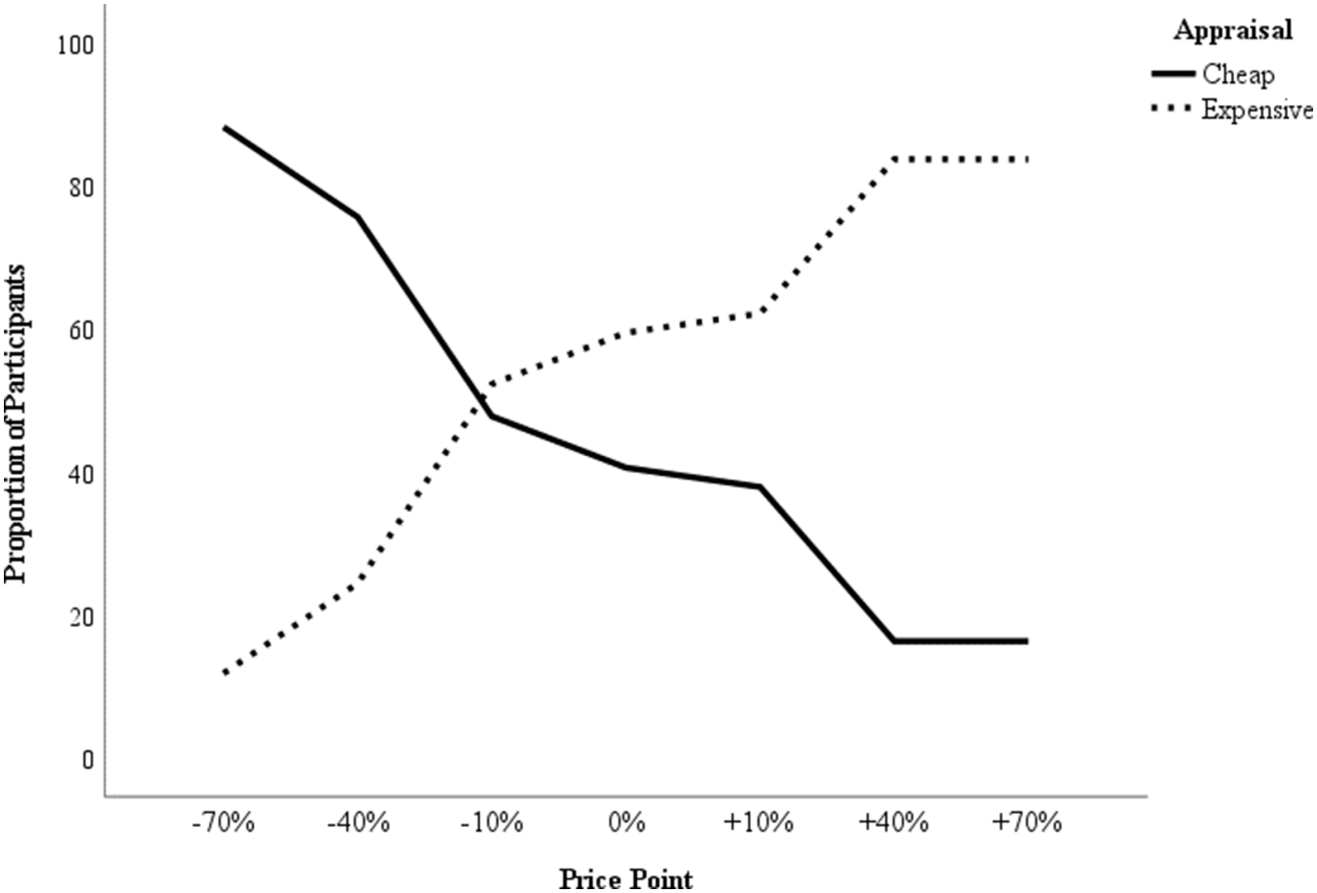
Using IMPACT data from Experiment 2 to derive a WTP estimate. The lines plot the proportion of participants that perceived each tested price point as cheap (constant line) or expensive (dotted line). The intersection of the two proportion lines marks the optimal price point.

The purpose of an effective WTP measure is to pin-point the optimal price point at which profit is maximised through ideal sales volume. Because a neutral score on the automatic price measure indicates that a price was appraised neither as cheap nor as expensive, Dezwaef et al. (2019) suggested that an effective strategy to derive the optimal price point might be to (1) apply direct measures to identify a narrow price interval within which the optimal price point lies, and then (2) use an automatic price measure to pin-point the optimal price. Elaborating on this approach, we suggest that the automatic price appraisals may be converted into WTP estimates following the same logic to that used by the Van Westendorp price sensitivity meter (Van Westendorp, 1976). Specifically, in the price sensitivity meter, respondents are asked to indicate at which price they would perceive a product or service to be (1) too cheap to be of quality, (2) a bargain, (3) starting to be expensive, and (4) too expensive to consider a purchase. Using a line graph that indicates the proportion of respondents on the Y-axis and the range of prices on the X-axis, this data is then plotted in four lines as the cumulative number of respondents who consider any given price on the X-axis as too cheap, cheap, expensive, or too expensive. Hereby, the intersection of the ‘too cheap’ and the ‘too expensive’ lines are considered the optimal price point, as it indicates the price at which dissatisfaction in either direction is minimised. Applying this logic to the automatic price appraisals, for each price point that was measured by the IMPACT we plotted the proportion of participants that perceived the price as cheap and the proportion of participants that perceived the price as expensive. The optimal price point is indicated by the intersection of these two proportions (see Figure 4 for an illustration of this approach).

Because the suggested approach of identifying a narrow price interval within which the optimal price point lies by using direct measures, and then applying an automatic price measure to pin-point the optimal price will, by default, exclude extreme prices, future research should establish (1) whether the IMPACT can reliably discern price appraisals when the increments between the tested price points are small (e.g., 5% increments) and (2) to what degree the WTP estimates derived from this combined approach translate into buying behaviour. Additionally, although our studies used implicit measures to assess WTP, other methods such as conjoint analysis are more commonly used in industry. Consequently, we note here that a direct comparison between the performance of implicit measures, such as the IMPACT, and those of methods more widely used in industry is needed to establish whether implicit methods actually have added value for estimating WTP. Such a comparison might also allow researchers to identify the circumstances and / or product types for which different methods for assessing WTP are particularly effective. Finally, in the current studies, there were (by design) too few trials of each trial type per product for D scores to be computed for individual products (as opposed to grouping the data preparation by price point). However, with both paradigms, it would be possible with the same number of trials to compare D scores by product if fewer products were tested. Investigating the exact differential added value of WTP estimates derived from implicit measures for various products or product categories may clarify which products are particularly well suited to implicit-measure derived WTP estimates.

## Data availability statement

The datasets presented in this study can be found in online repositories. The names of the repository/repositories and accession number(s) can be found at: https://osf.io/w4kvz/.

## Ethics statement

The studies involving humans were approved by the Research Code of Ethics of the Faculty of Economics and Business Administration, Ghent University. The studies were conducted in accordance with the local legislation and institutional requirements. The participants provided their written informed consent to participate in this study.

## Author contributions

DA: Conceptualization, Data curation, Formal analysis, Methodology, Project administration, Software, Visualization, Writing – original draft. AS: Conceptualization, Data curation, Funding acquisition, Methodology, Supervision, Writing – review & editing.

## Footnotes

^1^Note that the internal consistency we report here is likely confounded by the fact that scores were aggregated across products, as the setup in this experiment does not allow aggregation at the individual product level (we further address this issue in our Discussion). This type of aggregation likely resulted in an underestimation of the true internal consistency because different products at the same price point might be perceived differently by the participant (e.g., a small price reduction of 10% might be perceived as cheap for product A but not for product B). Accordingly, it can be expected that unambiguous prices (+/− 40% & +/− 70% i.e., prices that are more uniformly perceived as expensive or cheap across product types) should produce more consistent responses compared to ambiguous prices (0% and +/− 10%; i.e., prices that are less uniformly perceived as expensive or cheap across product type). Internal consistency scores obtained from computing separate scores for ambiguous price points (0% and +/− 10%; *R_sb_* = −0.03) and unambiguous price points (+/− 40% & +/− 70%; *R_sb_* = 0.55) were in line with this reasoning.

^2^As noted in Experiment 1, the internal consistency we report here is likely confounded by the fact that scores were aggregated across products, because different products at the same price point might be perceived differently by the participant (e.g., a small price reduction of 10% might be perceived as cheap for product A but not for product B). Accordingly, it can be expected that unambiguous prices (+/− 40% & +/− 70% i.e., prices that are more uniformly perceived as expensive or cheap across product types) should produce more consistent responses compared to ambiguous prices (0% and +/− 10%; i.e., prices that are less uniformly perceived as expensive or cheap across product type). As in Experiment 1, the internal consistency scores obtained from computing separate scores for ambiguous price points (0% and +/− 10%) and unambiguous price points (+/− 40% & +/− 70%) were again in line with this reasoning for both the IMPACT (ambiguous prices: *R_sb_* = −0.03; unambiguous prices: *R_sb_* = 0.51) and the TRC (ambiguous prices: *R_sb_* = −0.02; unambiguous prices: *R_sb_* = 0.40).
